# [Fam-] trastuzumab deruxtecan (DS-8201a)-induced antitumor immunity is facilitated by the anti–CTLA-4 antibody in a mouse model

**DOI:** 10.1371/journal.pone.0222280

**Published:** 2019-10-01

**Authors:** Tomomi Nakayama Iwata, Kiyoshi Sugihara, Teiji Wada, Toshinori Agatsuma

**Affiliations:** Oncology Research Laboratories I, R&D Division, Daiichi Sankyo Co., Ltd., Tokyo, Japan; University of Pécs Medical School, HUNGARY

## Abstract

[Fam-] trastuzumab deruxtecan (DS-8201a) is a HER2 (ERBB2)-targeting antibody-drug conjugate, composed of a HER2-targeting antibody and a topoisomerase I inhibitor, exatecan derivative, that has antitumor effects in preclinical xenograft models and clinical trials. Recently, [fam-] trastuzumab deruxtecan was reported to enhance antitumor immunity and was beneficial in combination with an anti–PD-1 antibody in a mouse model. In this study, the antitumor effect of [fam-] trastuzumab deruxtecan in combination with an anti–CTLA-4 antibody was evaluated. [Fam-] trastuzumab deruxtecan monotherapy had antitumor activity in an immunocompetent mouse model with EMT6 human HER2-expressing mouse breast cancer cells (EMT6-hHER2). [Fam-] trastuzumab deruxtecan in combination with the anti–CTLA-4 antibody induced more potent antitumor activity than that by monotherapy with either agent. The combination therapy increased tumor-infiltrating CD4^+^ and CD8^+^ T cells *in vivo*. Mechanistically, cured mice with treatment of [fam-] trastuzumab deruxtecan and an anti–CTLA-4 antibody completely rejected EMT6-mock cells similar to EMT6-hHER2 cells, and splenocytes from the cured mice responded to both EMT6-hHER2 and EMT6-mock cells as measured by interferon-gamma release. Taken together, these results indicate that antitumor immunity is induced by [fam-] trastuzumab deruxtecan and is facilitated in combination with anti–CTLA-4 antibody.

## Introduction

[Fam-] trastuzumab deruxtecan (DS-8201a) is a HER2-targeting antibody-drug conjugate (ADC) that is structurally composed of a humanized anti-human HER2 (anti-hHER2) antibody, an enzymatically-cleavable peptide-based linker, and an exatecan derivative topoisomerase I inhibitor [1,2]. [Fam-] trastuzumab deruxtecan has exhibited antitumor activity in a variety of mouse tumor models [1]. Moreover, in clinical studies, [fam-] trastuzumab deruxtecan has demonstrated antitumor effects in breast, gastric, gastro-esophageal, colorectal, salivary, and non-small cell lung cancer patients [3–6], including those previously treated with trastuzumab emtansine (T-DM1), an approved ADC composed of a tubulin polymerization inhibitor and an anti-hHER2 antibody. [Fam-] trastuzumab deruxtecan may work even in tumors expressing low levels of HER2, which cannot be treated by conventional HER2 therapies [3, 6].

Some chemotherapeutic agents can induce antitumoral immunity by mechanisms such as “immunogenic cell death” [7–9]. One mechanism by which chemotherapeutic agents activate the immune system is the activation of dendritic cells (DCs), which in turn activate T cells [7,8,10]. Intriguingly, the topoisomerase I inhibitors, irinotecan, topotecan, and camptothecin reportedly increase T cell-killing activity in patient-derived melanoma cell lines [11,12]; thus, topoisomerase I inhibitors have attracted increasing attention as immune stimulators.

Immune checkpoint inhibitors (ICIs), anti–PD-1/L1 antibodies, and an anti–CTLA-4 antibodies have been approved for clinical use and have remarkable clinical benefits [13–15]. Nevertheless, a limited number of patients have actually benefited from these therapies and new drugs or combination therapies with other drugs are required [16]. ADCs and chemotherapeutic agents including topoisomerase I inhibitors have been reported to have benefits when combined with ICIs in mouse models [10, 11, 17], with chemotherapeutic agent and ICI combinations also expected to have benefits in humans [18]. We previously reported that [fam-] trastuzumab deruxtecan enhances antitumor immunity in a mouse model by upregulating MHC class I expression on cancer cells and activation markers on DCs and increasing the prevalence of intra-tumor CD8^+^ T cells [19]. Furthermore, [fam-] trastuzumab deruxtecan was successfully combined with an anti–PD-1 antibody to improve antitumor activity in the mouse model [19].

The roles of the anti–CTLA-4 antibody are distinct from those of the anti–PD-1 antibody; the anti–CTLA-4 antibody restores T cell priming, whilst the anti–PD-1 antibody restores T cell effector function [9]. Here, we examined the benefits of [fam-] trastuzumab deruxtecan combined with the anti–CTLA-4 antibody in a mouse model, which has not previously been examined or reported.

## Materials and methods

### Antibodies and compounds

[Fam-] trastuzumab deruxtecan and its parental anti-HER2 antibody and isotype non-targeted control ADC (control ADC) were prepared as previously described [1, 2, 20]. The drug-to-antibody ratio was 7.7 ([fam-] trastuzumab deruxtecan) or 7.8 (control ADC) as determined by reverse phase chromatography. Anti–CTLA-4 antibody (clone 9D9, mouse IgG2b) was purchased from Bio X Cell. For flow cytometry analyses, fluorescein isothiocyanate labeled anti-hHER2 antibody (340553, Becton Dickinson), Pacific Blue labeled anti-mouse CD45 antibody (103126, BioLegend), phycoerythrin (PE) labeled anti-mouse T cell receptor (TCR) β antibody (109208, BioLegend), PerCP/Cy5.5 labeled anti-mouse CD4 antibody (100434, BioLegend), and PE-Cy 7 labeled anti-mouse CD8a antibody (552877, Becton Dickinson) were used.

### Flow cytometry

The LIVE/DEAD Fixable Near-IR Dead Cell Stain Kit (Thermo Fisher Scientific) was used to exclude dead cells. Cells were stained with antibodies as indicated. Flow cytometry data were acquired using the FACSCanto II device (Becton Dickinson) and data were analyzed with FlowJo 7.6.5 and 10.5.3 software (Becton Dickinson).

### Cell lines

The KPL-4 human breast cancer cell line was provided by Dr. Junichi Kurebayashi, Kawasaki Medical University (Japan). The CT26.WT and EMT6 mouse cancer cell lines (CRL 2638 and CRL 2755, respectively) were purchased from the American Type Culture Collection. Human *HER2* gene (NM_004448.3) inserted CT26.WT cells (CT26.WT-hHER2) were reported previously [19]. An empty vector (pLVSIN-EF1α, Clontech) or an expression vector inserted human *HER2* gene (NM_004448.3) was introduced into EMT6 cells using lentivirus (EMT6-mock and EMT6-hHER2, respectively). The cells were cultured in Waymouth's MB 752/1 medium supplemented with 15% fetal bovine serum and 500 μg/mL Geneticin (Thermo Fisher Scientific). Human HER2 expression was confirmed by flow cytometry.

### Mouse models, treatments, and analysis of intra-tumor cells

All animal experiments were conducted in an appropriate manner according to research ethics and due consideration was paid to the welfare of laboratory animals. Daiichi Sankyo has established in-house regulations (Detailed Regulations on Animal Experimentation) based on Japanese laws and guidelines, including the Act on Welfare and Management of Animals and the fundamental guidelines issued by the Ministry of Health, Labour, and Welfare and related activities. In accordance with these rules, we practice the 3Rs of animal research. All animal-use protocols must be reviewed by the company’s Institutional Animal Care and Use Committee (IACUC), and only approved protocols can be enacted. Moreover, researchers conducting animal research are required to undergo specialized education each year. To confirm that the animal care and use programs are in compliance with Japanese laws and guidelines, we conduct annual self-inspections and seek accreditation from third-party certifying organizations. The Shinagawa R&D Center has received and renewed its full accreditation from the AAALAC (Association for Assessment and Accreditation of Laboratory Animal Care) International.

Four or five mice were housed together in sterilized cages and maintained under specific pathogen-free conditions. The mice were euthanized with CO_2_ gas when they reached one of the endpoints (tumor volume exceeding 3000 mm^3^, 10% reduction in body weight, or clinical signs indicating that mice should be euthanized for ethical reasons–approved by the IACUC as a humane endpoint criteria). Five-week-old female BALB/cAnNCrlCrlj (BALB/c) mice were purchased from Charles River Laboratories Japan Inc. Six-week-old mice were subcutaneously (s.c) injected in the right flank with 1 × 10^6^ EMT6-hHER2 cells suspended in saline. Tumor volume was defined as 1/2 × length × width^2^. When the average volume of tumors reached approximately 100–200 mm^3^ (5 days after tumor inoculation), the mice were divided into control and treatment groups based on tumor volumes using the randomized block method, and treatment was initiated (treatment initiation day was defined as day 0). [Fam-] trastuzumab deruxtecan (10 mg/kg), anti-hHER2 antibody (10 mg/kg), control ADC (10 mg/mL), and anti–CTLA-4 antibody (5 mg/kg) were administered intravenously (i.v.) at a volume of 10 mL/kg. As a control, ABS buffer (10 mM acetate buffer and 5% sorbitol, pH 5.5) was administered at the same volume as the [fam-] trastuzumab deruxtecan. [Fam-] trastuzumab deruxtecan, anti-hHER2 antibody, and control ADC were administered on days 0 and 7. Anti–CTLA-4 antibody was administered on days 0, 3, 7, and 10.

For the re-challenge study, mice whose EMT6-hHER2 tumors had been cured (i.e. no measurable tumor for at least 4 weeks) by the combination treatment of [fam-] trastuzumab deruxtecan and the anti–CTLA-4 antibody were divided into two groups. The mice were then inoculated s.c. with 1 × 10^6^ EMT6-mock or 2 × 10^6^ EMT6-hHER2 cells into the left flank. Naïve (never tumor-inoculated and never treated) mice were also inoculated with each cell line for comparison.

For the flow cytometry analysis of T cells, larger tumors were used to obtain sufficient numbers of cells from the tumors. When the average volume of tumors reached approximately 300–400 mm^3^ (8 days after tumor inoculation), the mice were treated with vehicle, [fam-] trastuzumab deruxtecan (10 mg/kg, once, i.v.), anti–CTLA-4 antibody (5 mg/kg, days 0 and 2, i.v.), or the combination of [fam-] trastuzumab deruxtecan and anti–CTLA-4 antibody (treatment initiation day was defined as day 0). The mice were euthanized with CO_2_ inhalation on day 8 and tumors were cut into small pieces and dissociated with a Tumor Dissociation Kit (Miltenyi Biotec) using a gentleMACS Octo Dissociator with Heaters (Miltenyi Biotec). The resultant single cells were blocked with Mouse BD Fc Block reagent (Becton Dickinson) and stained with antibodies against mouse TCRβ, CD4, CD8, and CD45.

### Enzyme-linked immunospot (ELISPOT) assay

Splenocytes from EMT6-mock or EMT6-hHER2 tumor-bearing naïve mice and mice whose EMT6-hHER2 tumors had been cured by the combination of [fam-] trastuzumab deruxtecan and anti–CTLA-4 antibody treatment were subsequently co-cultured with EMT6-mock or EMT6-hHER2 cells at 37°C and 5% CO_2_ for 21 h in high-protein-binding polyvinylidene fluoride filter plates pre-coated with anti-mouse interferon-gamma (IFN-γ) monoclonal antibody (mIFNgp-1M/10, Cellular Technology Limited). IFN-γ secreted from the co-cultured cells was determined by a Murine IFN-γ Single-Color Enzymatic ELISPOT Assay using and ImmunoSpot S6 ENTRY Analyzer (Cellular Technology Limited) and ImmunoSpot 5.0 software (version 5.1.36, Cellular Technology Limited).

### Statistical analyses

**Fig 1:**
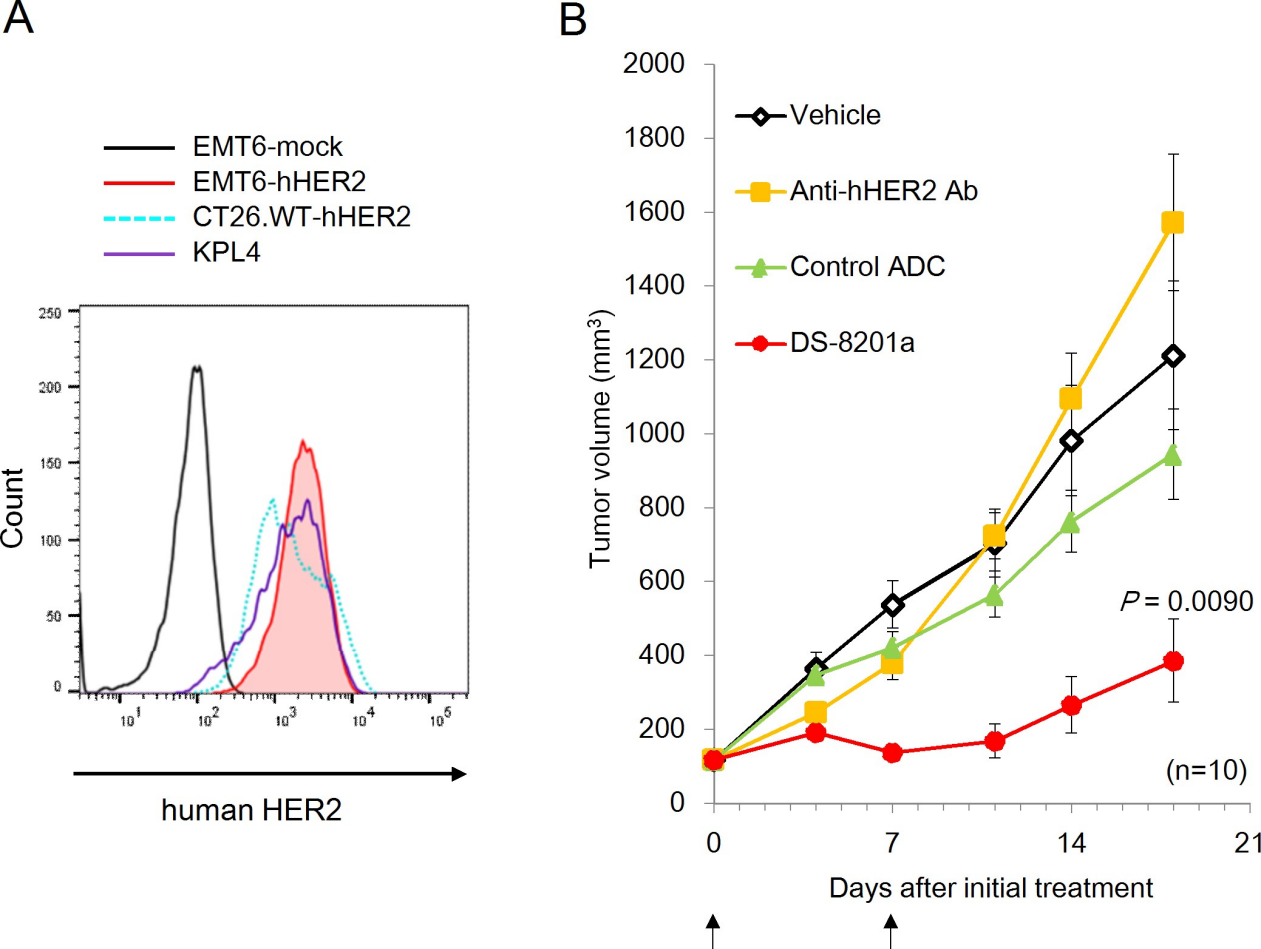
Antitumor effect of DS-8201a in EMT6-hHER2 syngeneic mouse model. **A,** Confirmed expression of human *HER2* gene exogenously introduced into EMT6 mouse breast cancer cells (EMT6-hHER2 cells) is displayed as a red filled peak. Flow cytometry analysis was conducted. **B,** Antitumor effect evaluated in an immunocompetent mouse model with EMT6-hHER2 cells. Anti-hHER2 antibody (Anti-hHER2 Ab, 10 mg/kg), control ADC (10 mg/kg), or [fam-] trastuzumab deruxtecan (DS-8201a) (10 mg/kg) were administrated at the time points indicated by the arrows. The graph shows mean tumor volumes and standard errors (n = 10 per group). Steel test was conducted for the statistical analysis of tumor volumes on day 18. *P <* 0.05 was considered statistically significant. Individual tumor growth curves are shown in S1 Fig.

**Fig 2:**
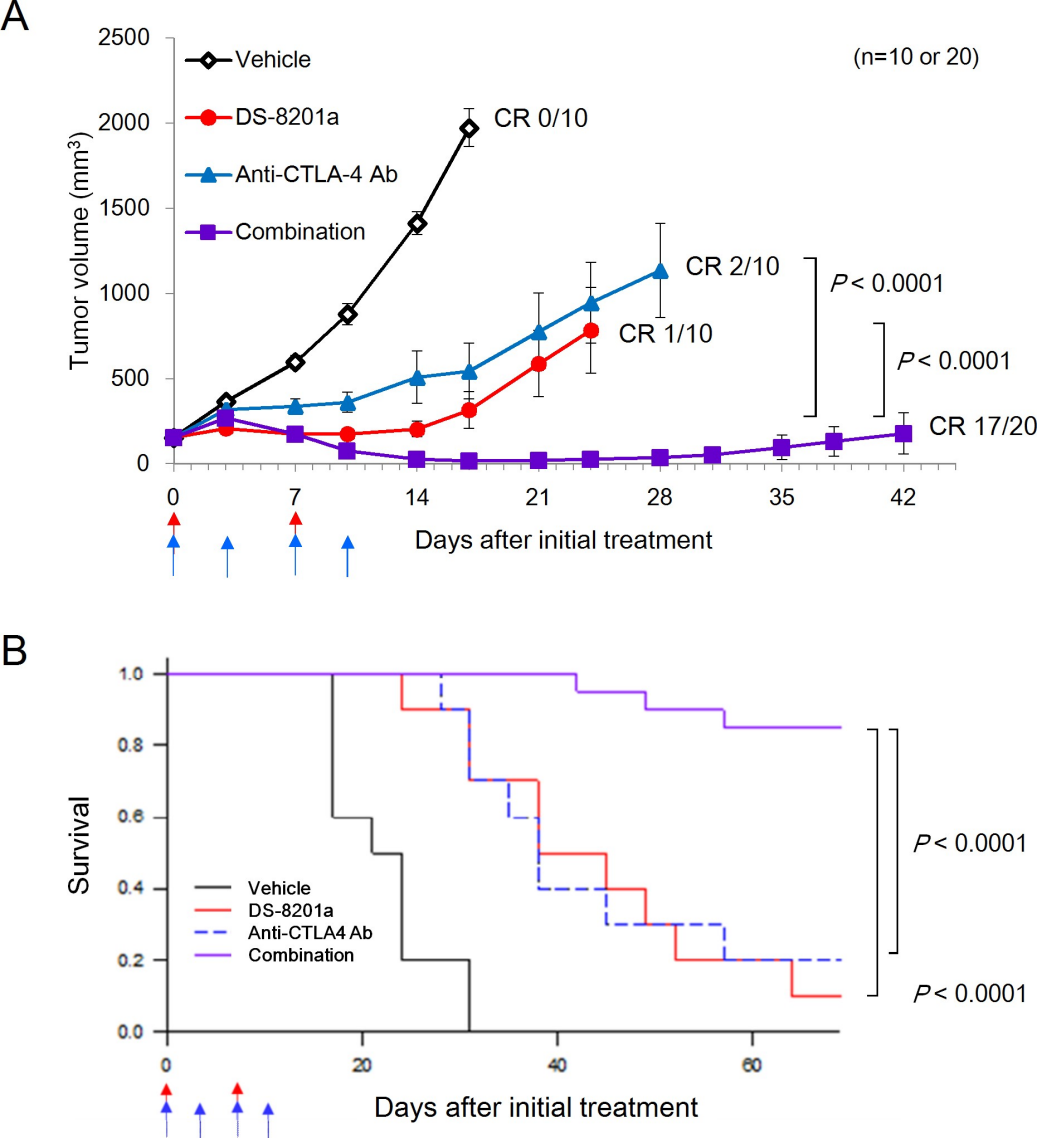
Combination effect of DS-8201a and anti–CTLA-4 antibody. Antitumor effect was evaluated in a syngeneic mouse model with EMT6-hHER2 cells. Effect of [fam-] trastuzumab deruxtecan (DS-8201a) (10 mg/kg) combined with anti–CTLA-4 antibody (anti–CTLA-4 Ab, 5 mg/kg) was compared with each agent applied individually. **A,** Graph showing mean tumor volumes and standard errors (n = 10 in the vehicle and monotherapy groups; n = 20 in the combination group). Compounds were administered at the time points indicated by the arrows (red; DS-8201a, blue; anti–CTLA-4 antibody). The tumor growth curves are interrupted in the middle of the study as some of mice had to be euthanized for an ethical reason before the study termination (Day 70). Complete response (CR) rates are shown in the graph. Steel test was conducted for the statistical analysis of tumor volumes on day 17. The vehicle-treated group was compared to the DS-8201a-treated group and the anti–CTLA-4 antibody-treated group (*P* = 0.0003, *P* = 0.0006, respectively). The combination (DS-8201a and anti–CTLA-4 antibody) group was also compared to the DS-8201a-treated group and the anti–CTLA-4 antibody-treated group (both *P* < 0.0001). **B,** Graph of the survival curve of the same experiment. The mice were euthanized when they reached the pre-defined endpoints summarized in the Materials and Methods. Survival was analyzed by the Kaplan-Meier method and groups were compared with the log-rank test. The vehicle-treated group was compared to the DS-8201a-treated group and the anti–CTLA-4 antibody-treated group (*P* = 0.0022 and *P* = 0.0012, respectively). The combination (DS-8201a and anti–CTLA-4 antibody) group was also compared to the DS-8201a-treated group and the anti–CTLA-4 antibody-treated group (both *P* < 0.0001).

**Fig 3:**
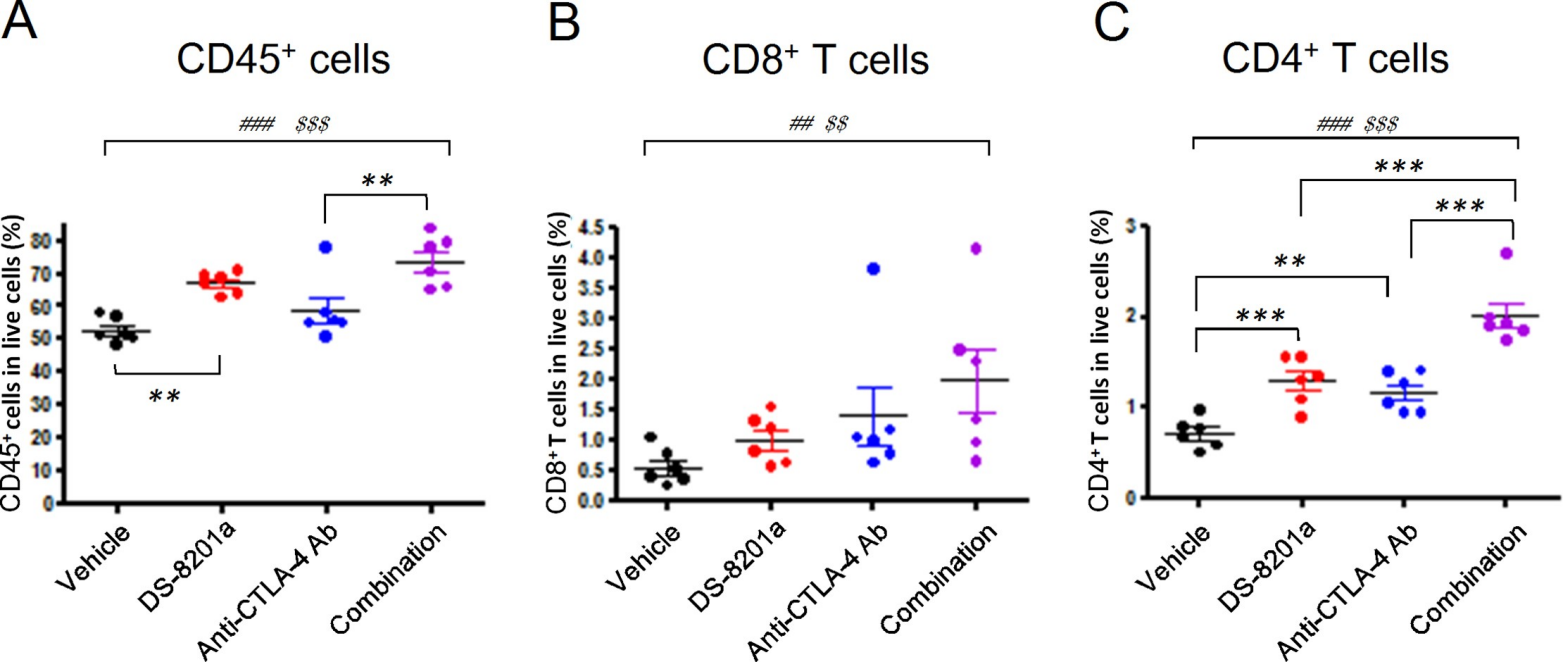
Increase in intra-tumor leukocytes and T cells. Effect of [fam-] trastuzumab deruxtecan (DS-8201a, 10 mg/kg) combined with anti–CTLA-4 antibody (anti–CTLA-4 Ab, 5 mg/kg) was examined in the immunocompetent mice subcutaneously inoculated with EMT6-hHER2 cells. The expression levels of CD45, TCRβ, CD8, and CD4 in cells from tumors were determined at day 8 by flow cytometry after the treatment. **A**, Percentages of CD45^+^ cells among live cells. **B**, Percentages of CD8^+^ T cells (CD45^+^ TCRβ^+^ CD8^+^ cells) among live cells. **C**, Percentages of CD4^+^ T cells (CD45^+^ TCRβ^+^ CD4^+^ cells) among live cells. The circles show individual data and the bars show means with standard errors (n = 6). Dunnett's multiple comparison test: **P < 0.01 and ***P < 0.001. Jonckheere's trend tests: ^##^*P* < 0.01 and ^###^*P* < 0.001 among the vehicle, DS-8201a, and combination groups, ^$ $^*P* < 0.01 and ^$ $ $^*P* < 0.001 among the vehicle, anti-CTLA-4 antibody, and combination groups. Details of statistical analyses are shown in S1 and S2 Tables.

**Fig 4:**
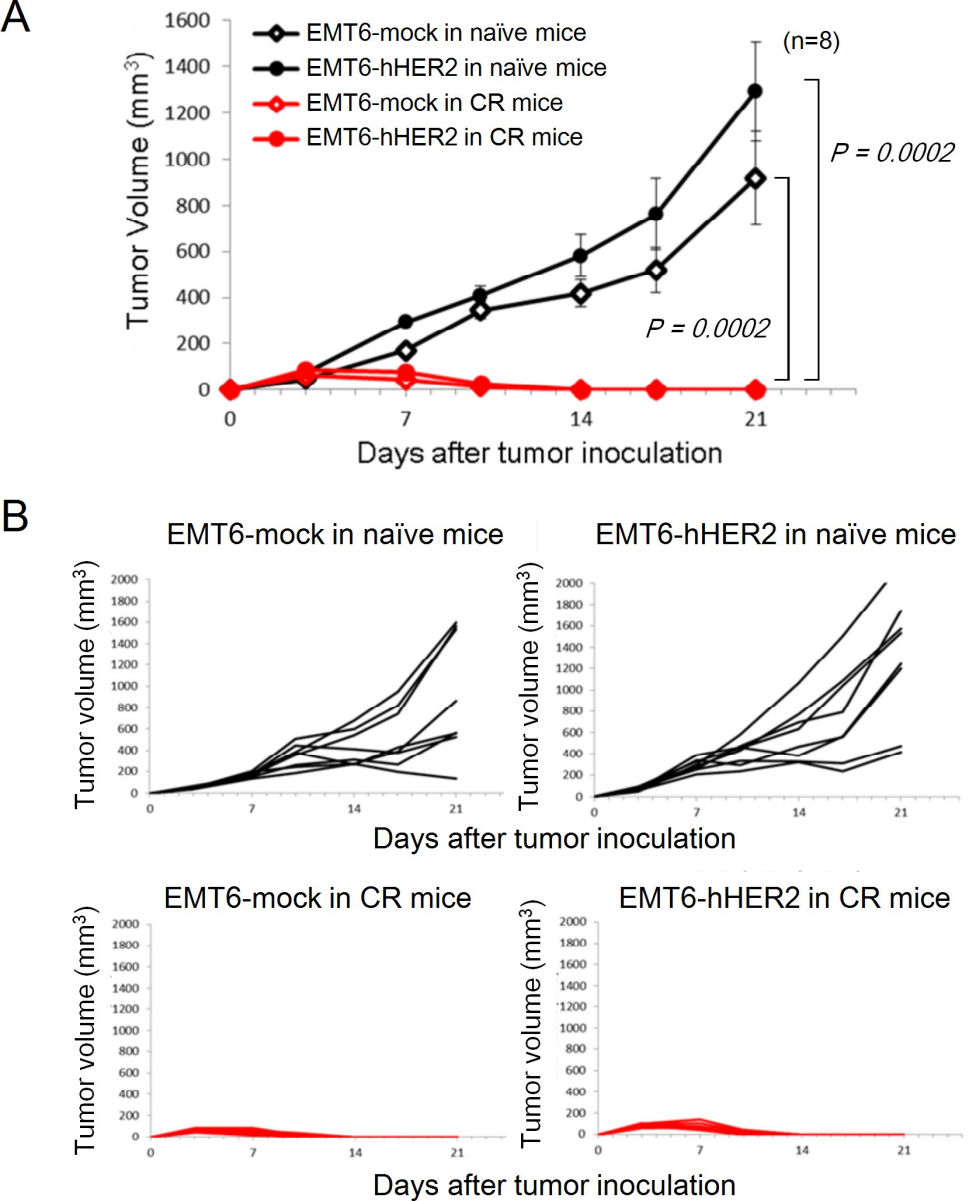
Antitumor immunity caused by combination of DS-8201a and anti–CTLA-4 antibody. Mice cured of EMT6-hHER2 tumors by treatment with the combination of [fam-] trastuzumab deruxtecan (DS-8201a, 10 mg/kg) and anti–CTLA-4 antibody (5 mg/kg) were re-challenged with EMT6-mock or EMT6-hHER2 cells and tumor growth was determined. As controls, naïve (never tumor-inoculated and never treated) mice were used. **A,** Mean tumor volumes and standard errors (n = 8) were shown. **B**, Spider plots of individual data. Wilcoxon rank sum test was conducted to compare naïve mice and cured mice.

**Fig 5:**
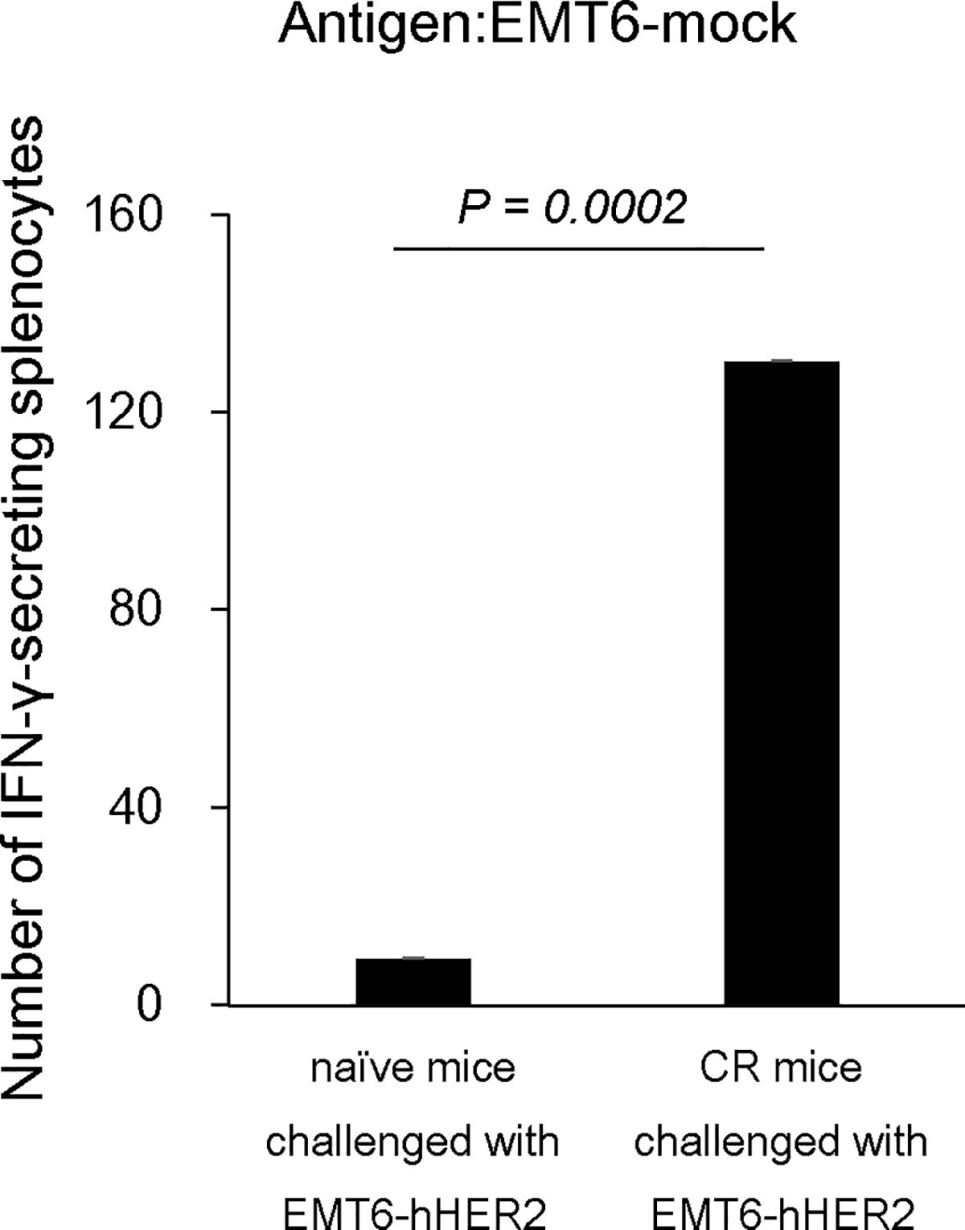
IFN-γ secretion by splenocytes from re-challenged mice. Splenocytes obtained from re-challenged mice previously cured of EMT6-hHER2 tumors using the combination treatment of [fam-] trastuzumab deruxtecan (DS-8201a) and anti–CTLA-4 antibody or splenocytes obtained from naïve mice challenged with EMT6-hHER2 cells were cultured with EMT6-mock cells. Immune cell reactivity to tumor cells was determined by determination of IFN-γ secretion using the ELISPOT assay. The number of spots represented the number of IFN-γ-secreting splenocytes. The plot shows the mean number of IFN-γ-secreting splenocytes and standard errors (n = 8). Wilcoxon rank sum test was conducted to compare naïve mice and cured mice.

Antitumor effects (Fig 1B, Fig 2A) were analyzed with Steel test. To analyze the survival curve of combination therapy, the day on which the estimated tumor volume exceeded 3000 mm^3^ (euthanized for an ethical reason) was set as the event occurrence date, and the period up to the immediately preceding measurement date was defined as the survival period. Kaplan-Meier analysis followed by the log-rank test were performed. Jonckheere-Terpstra trend tests were carried out to determine whether there was a statistically significant upward trend among the vehicle, monotherapy, and combination groups (Fig 3, S2 Fig and S1 Table). Dunnett’s multiple comparison tests were carried out to determine whether there were statistically significant differences between the vehicle and monotherapy groups or between the combination and monotherapy groups (Fig 3, S2 Fig and S2 Table). Post-hoc statistical analyses were conducted for Figs 1B, 2A and 3 and S2 Fig). Re-challenge study (Fig 4) and ELISPOT assays (Fig 5, S1 File) were analyzed with Wilcoxon rank sum test. A *P*-value < 0.05 was considered statistically significant. All statistical analyses except for Jonckheere-Terpstra trend tests were performed using SAS System Release 9.02.300 (SAS Institute Inc.). Jonckheere-Terpstra trend tests were performed using EXSUS ver.8.1 (CAC Croit, Tokyo, Japan) based on SAS release 9.4 (SAS Institute Inc.)

## Results

### Antitumor effect of [fam-] trastuzumab deruxtecan in mouse breast cancer model

Since the parental anti-hHER2 antibody of [fam-] trastuzumab deruxtecan does not bind to mouse ERBB2, we prepared a mouse cell line that stably expresses the human target gene [21–23] and used those cells to construct an immunocompetent mouse tumor model. The human *HER2* gene was introduced into EMT6 mouse breast cancer cells and the expression level of human HER2 in the EMT6-hHER2 cells was determined by flow cytometry analysis. Human HER2 was expressed on the EMT6-hHER2 cells at a level that was comparable to KPL-4 cells, a representative human HER2-positive (immunohistochemistry 3+ determined by Herceptest) cancer cell line [1, 24] and CT26.WT-hHER2 human HER2-expressing mouse cancer cell line [19] (Fig 1A). The EMT6-hHER2 cells were inoculated s.c. into BALB/c mice. After tumor engraftment was confirmed, mice with EMT6-hHER2 tumors were treated with vehicle, [fam-] trastuzumab deruxtecan (10 mg/kg), its parental anti-hHER2 antibody (10 mg/kg), or control ADC (10 mg/kg). [Fam-] trastuzumab deruxtecan significantly suppressed tumor growth compared to vehicle (*P* = 0.0090) at 18 days after treatment, while the parental anti-hHER2 antibody and isotype control ADC did not (Fig 1B, S1 Fig). These results suggested that in this model the antitumor effect of [fam-] trastuzumab deruxtecan depends on targeting antibody and its payload, but not on the anti-hHER2 antibody itself.

### Combination effect of [fam-] trastuzumab deruxtecan and anti–CTLA-4 antibody

[Fam-] trastuzumab deruxtecan reportedly enhances antitumor immunity and is beneficial when applied in combination with anti–PD-1 antibody in a mouse model [19]. To examine whether the benefit of [fam-] trastuzumab deruxtecan extends to another ICI, namely anti–CTLA-4 antibody, we examined the effect of this combination in a mouse model. Body weight reduction (>10%) was not observed in any mouse. At day 17, an antitumor effect was evident compared to vehicle for monotherapy of [fam-] trastuzumab deruxtecan (10 mg/kg, once a week, twice) or anti–CTLA-4 antibody (5 mg/kg, twice a week, 2 cycles) (*P* = 0.0003, *P* = 0.0006, respectively) (Fig 2A). The combined use of [fam-] trastuzumab deruxtecan and anti–CTLA-4 antibody produced an antitumor effect that was superior to monotherapy with [fam-] trastuzumab deruxtecan and anti–CTLA-4 antibody (both *P* < 0.0001) (Fig 2A). The survival rates for vehicle, [fam-] trastuzumab deruxtecan, anti–CTLA-4 antibody, and the combination at day 70 were 0%, 10%, 20%, and 85%, respectively (Fig 2B). [Fam-] trastuzumab deruxtecan or anti–CTLA-4 antibody monotherapy extended the overall survival time compared to vehicle (*P* = 0.0022 and *P* = 0.0012, respectively). Their combined use further increased the overall survival time compared with the monotherapy with [fam-] trastuzumab deruxtecan (*P* < 0.0001) and anti–CTLA-4 antibody (*P* < 0.0001) (Fig 2B). In addition, most of the mice treated with combination of [fam-] trastuzumab deruxtecan and anti–CTLA-4 antibody achieved a complete response (CR, 85%, Fig 2A). The results clearly demonstrated the benefit of [fam-] trastuzumab deruxtecan applied in combination with anti–CTLA-4 antibody.

### Increased intra-tumor T cells using the combination of [fam-] trastuzumab deruxtecan and anti–CTLA-4 antibody

To examine whether intra-tumoral lymphocytes especially T cells that are associated with significantly greater antitumor activity [25] were affected by these therapies, tumors of the EMT6-hHER2 immunocompetent mouse model were analyzed by flow cytometry. Eight days after each treatment ([fam-] trastuzumab deruxtecan; 10 mg/kg, once / anti–CTLA-4 antibody; 5 mg/kg, twice a week, 1 cycle / combination of [fam-] trastuzumab deruxtecan and anti–CTLA-4 antibody), the percentage of CD45^+^ cells, CD4^+^ T cells (CD45^+^TCRβ^+^CD4^+^ cells), and CD8^+^ T cells (CD45^+^TCRβ^+^CD8^+^ cells) in live cells were determined and post-hoc statistical analyses were conducted (Fig 3, S1 and S2 Tables).

Compared to the vehicle treatment group, the [fam-] trastuzumab deruxtecan monotherapy group and the combination group ([fam-] trastuzumab deruxtecan and anti–CTLA-4 antibody) exhibited an upward trend in the percentages of CD45^+^ cells, CD4 T^+^ cells, and CD8^+^ T cells in the live cells (Fig 3, S1 Table; Jonckheere-Terpstra trend test). The same trend was observed in the percentages of CD45^+^, CD4^+^ T cells, and CD8^+^ T cells in live cells when the vehicle treatment group was compared with the anti–CTLA-4 antibody monotherapy group and the combination group (Fig 3, S1 Table; Jonckheere-Terpstra trend test). The upward trend in CD4^+^ T cells and CD8^+^ T cells was also confirmed by the percentage of CD4^+^ T cells and CD8^+^ T cells in the CD45^+^ cells in the tumors (S2 Fig, S1 Table). Moreover, Dunnett’s multiple comparison tests between the vehicle group and each monotherapy group indicate that the percentages of CD4^+^ T cells in the live cells and in the CD45^+^ cells were significantly increased by [fam-] trastuzumab deruxtecan and anti–CTLA-4 antibody treatment (Fig 3C, S2 Table, S2 Fig; Dunnett’s test). In addition, the combination group exhibited increased numbers of CD4^+^ T cells in the live cells and CD45^+^ cells compared with each monotherapy group (Fig 3C, S2 Table, S2 Fig; Dunnett’s test).

These results suggested that the combination therapy of [fam-] trastuzumab deruxtecan and anti–CTLA-4 antibody increases intra-tumor T cells.

### Combination of [fam-] trastuzumab deruxtecan and anti–CTLA-4 antibody enhances adaptive immunity

Since the combination of [fam-] trastuzumab deruxtecan and anti–CTLA-4 antibody produced an 85% CR in the treated mice (Fig 2A and 2B) and increased leukocytes and T cells were observed in the combination treatment (Fig 3, S2 Fig), we examined whether the combination treatment of [fam-] trastuzumab deruxtecan and anti–CTLA-4 contributed further to immune memory formation. The CR mice were re-challenged with EMT6-hHER2 or EMT6-mock cells. As controls, naïve mice that had never been inoculated with tumor cells and never treated were also challenged. EMT6-hHER2 and EMT6-mock cells were rejected in the CR mice, while those tumors grew normally in naïve mice (Fig 4A and 4B) (both *P* = 0.0002, Wilcoxon rank sum test). Splenocytes from these mice were obtained at the endpoint of the re-challenge study and co-cultured with EMT6-hHER2 or EMT6-mock cells. Secretion of IFN-γ was detected with the ELISPOT assay. Splenocytes from naïve mice challenged with EMT6-hHER2 or EMT6-mock cells minimally responded to tumor cells when they were co-cultured with EMT6-hHER2 and EMT6-mock cells (Fig 5, S3 Fig). By contrast, splenocytes from CR mice re-challenged with EMT6-hHER2 or EMT6-mock cells more significantly responded (Fig 5, S3 Fig). These results suggested that the combination of [fam-] trastuzumab deruxtecan and anti–CTLA-4 antibody produced an immune memory that recognized antigen(s) in EMT6 cells.

## Discussion

Immunotherapy is now recognized as the fourth pillar of cancer therapy, preceded by surgery, radiation, and chemotherapy/molecular targeted therapy [26]. The strengths of immunotherapy include its durable effects and survival benefits. Mechanistically, the immune system memorizes and surveys tumor antigens for a long time [27]; thus, a long-lasting effect is expected. Previously, we reported the importance of [fam-] trastuzumab deruxtecan in the activation of tumor cell immunity and the formation of immune memory in a CT26.WT-hHER2 syngeneic mouse model [19]. In the mouse model, [fam-] trastuzumab deruxtecan induced the expression of dendritic cell activation makers, upregulation of MHC class I in tumor cells, activation of CD8^+^ T cells in tumors, and formation of immune memory. The activation of dendritic cells promotes T cell activation, whilst MHC class I upregulation promotes tumor recognition by T cells since antigen presentation by MHC class I activates T cell receptors and thus T cell immunity [28]. The *in vivo* efficacy of [fam-] trastuzumab deruxtecan was inhibited by the depletion of CD8^+^ T cells in the model [19], suggesting that CD8^+^ T cells play an important role in antitumor activity. Moreover, the combination therapy of [fam-] trastuzumab deruxtecan and the anti–PD-1 antibody was more effective than either monotherapy [19]. In this study, we examined whether the immune-activating effect and antitumor activity of [fam-] trastuzumab deruxtecan was facilitated by the anti–CTLA-4 antibody, another type of ICI.

We demonstrated the substantial benefits of [fam-] trastuzumab deruxtecan combined with the anti–CTLA-4 antibody in the EMT-hHER2 mouse model; the combination produced an antitumor effect that significantly exceeded the effect produced by each monotherapy and produced a high CR. Furthermore, the mice cured by the combination therapy entirely rejected not only re-challenged EMT6-hHER2, but also EMT6-mock cells (Fig 4). The rejection of EMT6-mock cells in the CR mice suggests that the tumor rejection was not solely dependent on the exogenous human HER2 antigen (the target of [fam-] trastuzumab deruxtecan) and that the immune system recognized other tumor antigen(s) in EMT6. This is very attractive since the suppression of tumors by the immune system may continue even after tumor cells lose HER2 expression. These results suggest that the combination treatment can induce substantial immune activation and memory formation to eliminate tumor cells.

There are concerns over the cytotoxicity of chemotherapeutic agents to immune cells. In the case of [fam-] trastuzumab deruxtecan, it did not reduce the antitumor effect of the anti–CTLA-4 antibody, instead exhibiting a combined antitumor benefit. Furthermore, we previously reported that [fam-] trastuzumab deruxtecan confers a benefit when applied in combination with the anti–PD-1 antibody in another mouse model [19]. These results suggests that [fam-] trastuzumab deruxtecan does not have an obvious negative impact on immune cells that play important roles in antitumor immunity and that it can be combined with a variety of ICIs in mouse models.

Although both the anti–CTLA-4 and anti–PD-1/L1 antibodies are categorized as ICIs, their mechanisms of action differ [9,29,30] and would therefore lead to different outcomes [29]. Interestingly, it has been reported that the benefit of the anti–PD-1 antibody may not be as durable as that of the anti–CTLA-4 antibody at least in mice [29]. In a preclinical mouse model, the anti–CTLA-4 antibody induced a superior antitumor memory response during tumor re-challenge than the anti–PD-1 antibody [29]. These results suggest that the combination of [fam-] trastuzumab deruxtecan and the anti–CTLA-4 antibody could reduce relapse and increase survival; however, further elucidation and detailed analyses are required to confirm this hypothesis.

The combination of the anti–PD-1 and anti–CTLA-4 antibodies has exhibited superior antitumor effects to either monotherapy in mouse tumor models. The combination of nivolumab and ipilimumab has superior antitumor effects to either monotherapy in patients with certain tumor types and is indicated for the treatment of unresectable or metastatic melanoma, intermediate- or poor-risk patients with previously untreated advanced renal cell carcinoma, and microsatellite instability-high (MSI-H) or mismatch repair deficient (dMMR) metastatic colorectal cancer that has progressed following treatment with a fluoropyrimidine, oxaliplatin, and irinotecan [31]. Since our studies indicate the benefits of the combination of [fam-] trastuzumab deruxtecan with anti–CTLA-4 or anti–PD-1 antibodies, increased antitumor effects of [fam-] trastuzumab deruxtecan might be expected for [fam-] trastuzumab deruxtecan combined with both the anti–PD-1/L1 and anti–CTLA-4 antibodies.

Interestingly, treatment with [fam-] trastuzumab deruxtecan monotherapy results in a durable survival benefit and/or stable disease, even in some patients with tumors [4]; for example, a prolonged response (> 21.8 months) was observed in patients with breast cancer after receiving T-DM1 treatment. These results suggest that [fam-] trastuzumab deruxtecan might enhance tumor immunity even in patients with tumors, although further studies are necessary to confirm this. In addition, the combination of [fam-] trastuzumab deruxtecan with ICIs may increase antitumor immunity and produce a long-term benefit. The combination therapy of exatecan derivative-based ADCs, such as [fam-] trastuzumab deruxtecan, with ICIs may provide new opportunities for treating patients with tumors.

## Supporting information

S1 Table Jonckheere-Terpstra trend test. (PDF)

S2 Table Dunnett’s multiple comparison test. (PDF)

S1 Fig Individual spider plots for Fig 1B. (PDF)

S2 Fig Increase in intra-tumor T cells in CD45^+^ cells. (PDF)

S3 Fig IFN-γ secretion by splenocytes from re-challenged mice. (PDF)

S1 File Individual ELISPOT data. (PDF)
