# Supplementary material for: [Fam-] trastuzumab deruxtecan (DS-8201a)-induced antitumor immunity is facilitated by the anti–CTLA-4 antibody in a mouse model
Source: PLoS One. 2019 Oct 1;14(10):e0222280. doi: 10.1371/journal.pone.0222280 (PMC6772042; doi:10.1371/journal.pone.0222280)
Supplement: S1 Table — (PDF) [file pone.0222280.s001.pdf]

S1 Table. Jonckheere-Terpstra trend test

Cell population	Group	P value	Mark
$CD45^+$ cells in live cells	Vehicle, DS-8201a, Combination	0.0001	###
	Vehicle, Anti–CTLA-4 Ab, Combination	0.0007	$$$
$CD4^+$ T cells in live cells	Vehicle, DS-8201a, Combination	0.0000	###
	Vehicle, Anti–CTLA-4 Ab, Combination	0.0000	$$$
$CD8^+$ T cells in live cells	Vehicle, DS-8201a, Combination	0.0014	##
	Vehicle, Anti–CTLA-4 Ab, Combination	0.0051	$$
$CD4^+$ T cells in $CD45^+$ cells	Vehicle, DS-8201a, Combination	0.0000	###
	Vehicle, Anti–CTLA-4 Ab, Combination	0.0000	$$$
$CD8^+$ T cells in $CD45^+$ cells	Vehicle, DS-8201a, Combination	0.0116	#
	Vehicle, Anti–CTLA-4 Ab, Combination	0.0150	$

Post-hoc statistical analyses (Jonckheere-Terpstra trend tests) were conducted to determine whether there were statistically significant upward trend in the percentage of tumor infiltrating cells among the vehicle, monotherapy, and combination groups (Fig 3, S2 Fig).

Combination: DS-8201a and anti–CTLA-4 Ab

$^{\#}P < 0.05$, $^{\#\#}P < 0.01$, $^{\#\#\#}P < 0.001$

$^{\$}P < 0.05$, $^{\$\$}P < 0.01$, $^{\$\$\$}P < 0.001$
