# Supplementary material for: [Fam-] trastuzumab deruxtecan (DS-8201a)-induced antitumor immunity is facilitated by the anti–CTLA-4 antibody in a mouse model
Source: PLoS One. 2019 Oct 1;14(10):e0222280. doi: 10.1371/journal.pone.0222280 (PMC6772042; doi:10.1371/journal.pone.0222280)
Supplement: S2 Table — (PDF) [file pone.0222280.s002.pdf]

S2 Table. Dunnett's multiple comparison test

Cell population	Control group	Comparative group	P value	Mark
$CD45^+$ cells in live cells	vehicle	DS-8201a	0.0024	**
		Anti–CTLA-4 Ab	0.1970	
	Combination	DS-8201a	0.2371	
		Anti–CTLA-4 Ab	0.0059	**
$CD4^+$ T cells in live cells	vehicle	DS-8201a	0.0007	***
		Anti–CTLA-4 Ab	0.0045	**
	Combination	DS-8201a	0.0007	***
		Anti–CTLA-4 Ab	0.0002	***
$CD8^+$ T cells in live cells	vehicle	DS-8201a	0.4894	
		Anti–CTLA-4 Ab	0.1232	
	Combination	DS-8201a	0.2165	
		Anti–CTLA-4 Ab	0.5407	
$CD4^+$ T cells in $CD45^+$ cells	vehicle	DS-8201a	0.0112	*
		Anti–CTLA-4 Ab	0.0042	**
	Combination	DS-8201a	0.0022	**
		Anti–CTLA-4 Ab	0.0051	**
$CD8^+$ T cells in $CD45^+$ cells	vehicle	DS-8201a	0.5836	
		Anti–CTLA-4 Ab	0.0694	
	Combination	DS-8201a	0.2382	
		Anti–CTLA-4 Ab	0.7955	

Post-hoc statistical analyses (Dunnett's multiple comparison tests) were conducted to determine whether there were statistically significant differences in the percentage of tumor infiltrating cells among the vehicle, combination, and monotherapy groups (Fig 3, S2 Fig).

Combination: DS-8201a and anti–CTLA-4 Ab

*$P < 0.05$, **$P < 0.01$, ***$P < 0.001$
