# Supplementary figures and images for: [Fam-] trastuzumab deruxtecan (DS-8201a)-induced antitumor immunity is facilitated by the anti–CTLA-4 antibody in a mouse model
Source: PLoS One. 2019 Oct 1;14(10):e0222280. doi: 10.1371/journal.pone.0222280 (PMC6772042; doi:10.1371/journal.pone.0222280)

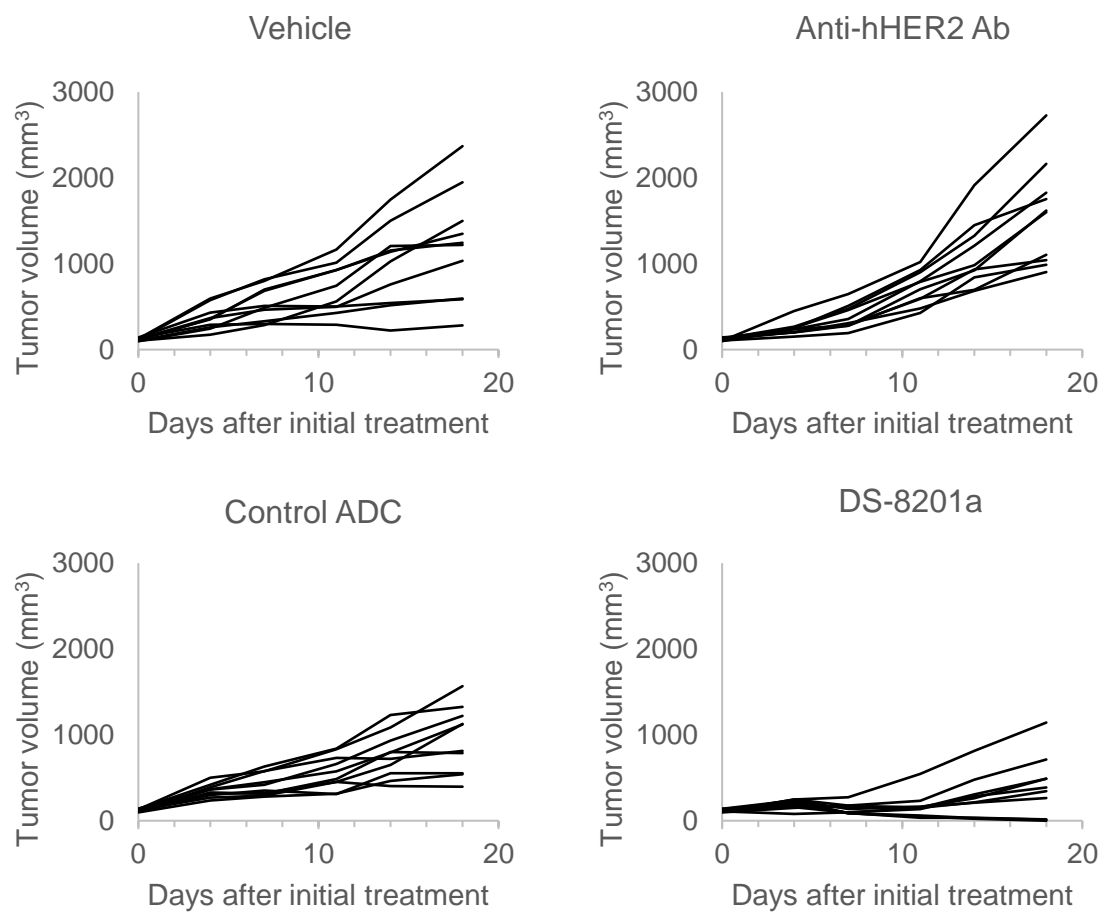

**S1 Figure. Individual spider plots for Fig. 1B**

Supplement: S1 Fig — (PDF) [file pone.0222280.s003.pdf]
