# Supplementary material for: [Fam-] trastuzumab deruxtecan (DS-8201a)-induced antitumor immunity is facilitated by the anti–CTLA-4 antibody in a mouse model
Source: PLoS One. 2019 Oct 1;14(10):e0222280. doi: 10.1371/journal.pone.0222280 (PMC6772042; doi:10.1371/journal.pone.0222280)
Supplement: S2 Fig — (PDF) [file pone.0222280.s004.pdf]

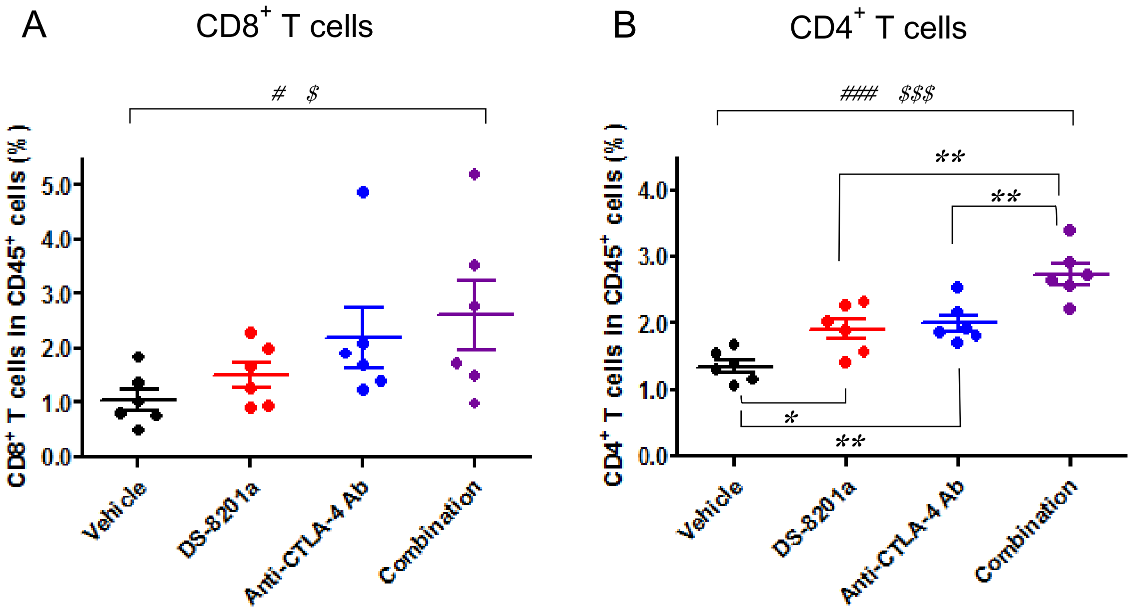


**S2 Figure. Increase in intra-tumoral T cells in $CD45^+$ cells**

The effect of [fam-] trastuzumab deruxtecan (DS-8201a, 10 mg/kg) combined with the anti–CTLA-4 antibody (anti–CTLA-4 Ab, 5 mg/kg) was examined in immunocompetent mice subcutaneously inoculated with EMT6-hHER2 cells. The expression levels of CD45, TCRβ, CD8, and CD4 in cells from tumors were determined on day 8 after treatment by flow cytometry. **A.** Percentage of $CD8^+$ T cells ($CD45^+$ $TCR\beta^+$ $CD8^+$ cells) among the $CD45^+$ cells. **B**. Percentage of $CD4^+$ T cells ($CD45^+$ $TCR\beta^+$ $CD4^+$ cells) among the $CD45^+$ cells. The circles show individual data points and the bars indicate the means and standard error (n = 6). Dunnett's multiple comparison test: $^{*}P < 0.05$ and $^{**}P < 0.01$. Jonckheere-Terpstra trend tests: $^{\#}P < 0.05$ and $^{\#\#\#}P < 0.001$ among the vehicle, DS-8201a, and combination groups. $^{\$}P < 0.05$ and $^{\$\$\$}P < 0.001$ among the vehicle, anti–CTLA-4, and combination groups. Details of statistical analyses are shown in S1 and S2 Tables.
