# Supplementary material for: [Fam-] trastuzumab deruxtecan (DS-8201a)-induced antitumor immunity is facilitated by the anti–CTLA-4 antibody in a mouse model
Source: PLoS One. 2019 Oct 1;14(10):e0222280. doi: 10.1371/journal.pone.0222280 (PMC6772042; doi:10.1371/journal.pone.0222280)
Supplement: S3 Fig — (PDF) [file pone.0222280.s005.pdf]

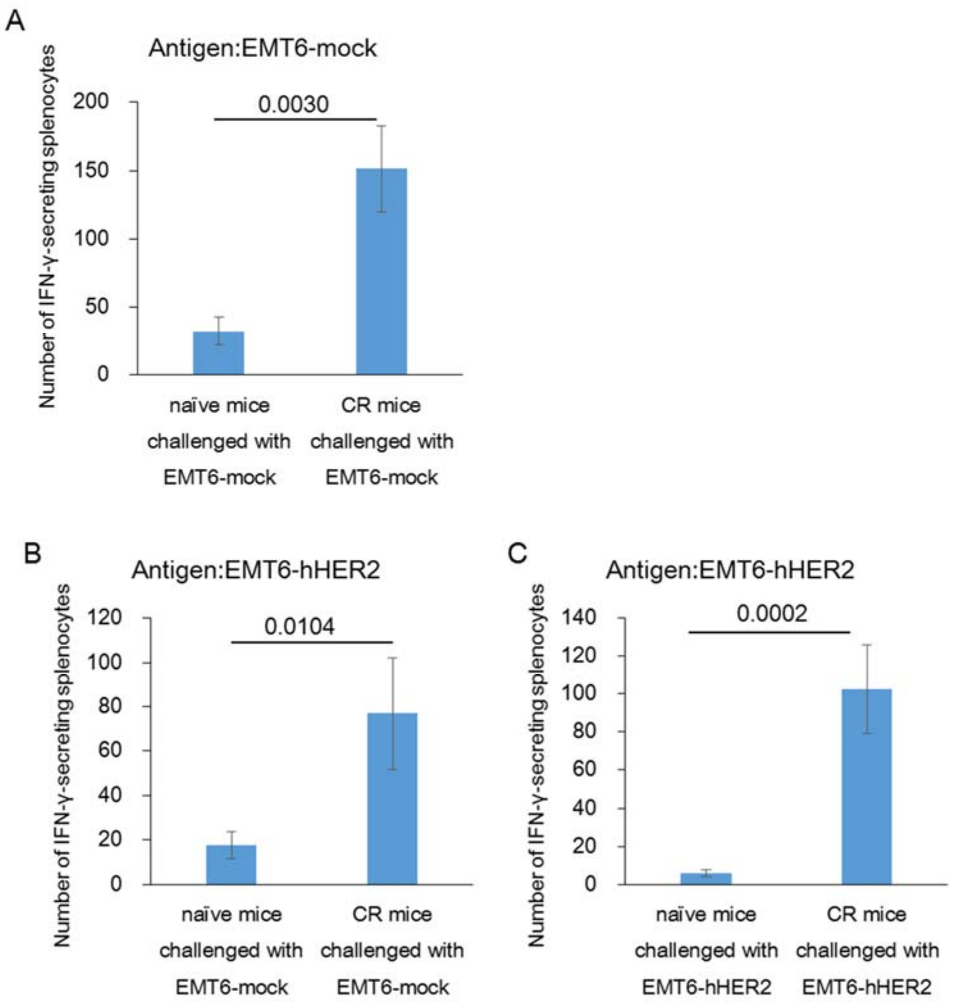

S3 Figure. IFN-γ secretion by splenocytes from re-challenged mice

Splenocytes were obtained from re-challenged mice previously cured of EMT6-hHER2 tumors using a combination of [fam-] trastuzumab deruxtecan and anti-CTLA4 antibody with EMT6-mock or EMT6-hHER2 cells, and naïve mice challenged with EMT6-mock or EMT6-hHER2 cells. IFN-γ secretion was examined by ELISPOT assays. The number of spots represented the number of IFNγ-secreting splenocytes. Splenocytes from mice challenged with EMT6-mock cells were cultured with EMT6-mock (**A**) or EMT6-hHER2 cells (**B**), and immune cell reactivity to tumor cells was determined by IFN-γ secretion. Splenocytes from the mice challenged with EMT6-hHER2 cells were cultured with EMT6-hHER2 cells (**C**), and immune cell reactivity to tumor cells was determined by IFN-γ secretion. Graphs show the mean number of IFN-γ-secreting splenocytes and standard errors (n = 8). Wilcoxon rank sum test was used to compare naïve and cured mice.
