# Supplementary material for: [Fam-] trastuzumab deruxtecan (DS-8201a)-induced antitumor immunity is facilitated by the anti–CTLA-4 antibody in a mouse model
Source: PLoS One. 2019 Oct 1;14(10):e0222280. doi: 10.1371/journal.pone.0222280 (PMC6772042; doi:10.1371/journal.pone.0222280)
Supplement: S1 File — (PDF) [file pone.0222280.s006.pdf]

A. Individual number of spots

Group 1. EMT6-mock in naïve mice

	Antigen	
Individual No.	EMT6-mock	EMT6-hHER2
1	22.5	22.5
2	81.5	52.0
3	9.5	7.0
4	75.5	14.0
5	13.5	2.5
6	11.0	5.0
7	18.5	6.5
8	25.0	31.0

Group 2. EMT6-hHER2 in naïve mice

	Antigen	
Individual No.	EMT6-mock	EMT6-hHER2
1	6.0	4.5
2	5.5	2.5
3	27.5	15.0
4	8.5	0.0
5	9.5	0.5
6	2.0	3.0
7	4.5	9.5
8	12.0	12.0

Group 3. EMT6-mock in CR mice

	Antigen	
Individual No.	EMT6-mock	EMT6-hHER2
1	115.5	73.0
2	79.0	38.5
3	52.5	20.0
4	136.5	18.0
5	68.5	40.0
6	235.0	79.5
7	270.5	111.5
8	252.0	234.5

Group 4. EMT6-hHER2 in CR mice

	Antigen	
Individual No.	EMT6-mock	EMT6-hHER2
1	68.0	78.0
2	41.5	29.0
3	60.0	35.5
4	358.5	230.5
5	112.0	77.5
6	107.5	88.0
7	191.5	147.5
8	103.5	135.0

CR, complete response.

B. The mean number of spots and standard errors of each group

		Antigen	
Group	mice	EMT6-mock	EMT6-hHER2
1	EMT6-mock in naïve mice	32 ± 10	18 ± 6
2	EMT6-hHER2 in naïve mice	9 ± 3	6 ± 2
3	EMT6-mock in CR mice	151 ± 31	77 ± 25
4	EMT6-hHER2 in CR mice	130 ± 36	103 ± 23

CR, complete response.
